# Objective Structured Clinical Examination Skill Station Is Effective for Assessing Acute Opioid Overdose Resuscitation

**DOI:** 10.7759/cureus.11511

**Published:** 2020-11-16

**Authors:** Matthew P Abrams, Martin Klapheke

**Affiliations:** 1 Psychiatry, University of Central Florida College of Medicine, Orlando, USA

**Keywords:** education and training of medical students and doctors (specialist and phd)), addiction psychiatry, chronic pain management, opioid withdrawal, opioid use disorders

## Abstract

Introduction

In recent decades, deaths related to heroin, illicit fentanyl, and prescription opioids have risen in the United States. Utilizing new clinical guidelines and non-prescription naloxone, we aimed to develop a competency-based assessment for clinical skills in opioid overdose resuscitation outside of the hospital setting.

Methods

An assessment of opioid resuscitation skills, consisting of an Objective Structured Clinical Examination (OSCE) skill station-utilizing a simulation mannequin and a standardized patient portraying the patient’s relative-followed by a facilitated individual debrief, was added to the fourth year Psychiatry Boot Camp for students entering a psychiatry residency. A survey was given to students to assess the OSCE’s believability, value, and impact on confidence in managing out-of-hospital overdose.

Results

Following the OSCE, 2017-2019 graduating students entering a psychiatry residency (N=10) all agreed or strongly agreed that the OSCE “was realistic and believable” and “was valuable as an educational tool. Most either agreed (N=7) or strongly agreed (N=1) they felt confident of their skill in managing out-of-hospital opioid overdose. A small number (n=2) were neutral in the confidence of their skill in managing out-of-hospital opioid overdose.

Discussion

Based on early medical student feedback (n=10), this OSCE skill station provides a promising competency-based assessment for opioid overdose resuscitation outside of the hospital setting. Its use could be expanded to other medical disciplines in undergraduate and graduate education.

## Introduction

The need for strengthening undergraduate medical training on opioid misuse is well-established. According to the Centers for Disease Control (CDC), about 47,000 people die in the United States from an opioid overdose each year, and the opioid crisis is continuing to worsen [1,2]. The U.S. Department of Health and Human Services now considers the opioid crisis a public health emergency [3]. Contributing to this public health crisis is a rise in illicitly made fentanyl [3]. Consequently, many medical students, residents (e.g. psychiatry, emergency medicine), among others, are likely to encounter opioid overdose patients brought to their medical centres and even outside hospital settings. 

According to the most recent clinical guidelines, the preferred treatment for a known or suspected opioid overdose with respiratory or central nervous system depression is naloxone [4,5]. Timely and proper administration of naloxone has been demonstrated to be life-saving in the treatment of opioid overdose [6,7]. Furthermore, the Department of Health and Human Services has identified “better targeting of overdose-reversing drugs” as one of its five major points in its strategy for combatting the opioid epidemic [3]. Therefore, it is critical that medical students and physicians alike are trained on how to properly administer naloxone and provide proper opioid resuscitation treatments. Existing literature suggests that currently some physicians lack comfort with opioid overdose resuscitation techniques and lack adequate training [8,9], possibly explaining why physicians have also been shown to be under-prescribing naloxone prevention treatments [10]. To help address the gap between naloxone access and effective naloxone use by healthcare providers, a 2020 study outlined the development of a rapid naloxone administration workshop for healthcare trainees; it aimed to provide reliable, standardized curricula on how to administer this life-saving medication effectively [11].

The literature on OSCEs or simulations involving opioid overdose is limited. In search of medical education databases, only one simulation article specifically focused on opioid overdose was found [12]. However, the article, published in 2017, involved a simulation that applied only to emergency medicine residents and pertained to the management of flash pulmonary edema, a rare complication of naloxone treatment for opioid overdose, and use of extracorporeal membrane oxygenations, rather than focusing on general opioid overdose treatments [13]. In general, naloxone for opioid overdose has become core knowledge for many specialities, yet formal literature is scant on studies of educational methods for medical trainees regarding opioid use disorder assessment and management [14,15,16]. This paper is one of the first to study an OSCE station formally, but the authors acknowledge that teaching points and activity associated with this work are reflected in many simulation scenarios and case-based discussions. 

## Materials and methods

Development

The OSCE was selected as the method by which to assess the application of this clinical knowledge as it is a well-established tool and it is well-suited for the evaluation of learners’ clinical skills in the setting of unpredictable patient behavior (such as respiratory depression from opioid overdose) [17]. Specifically, the OSCE is a versatile, reproducible, multipurpose evaluative tool that assesses competency, based on objective testing through direct observation that allows for uniform testing of students for a wide range of clinical skills and communication skills [18].

This opioid overdose scenario was a clinical skill station performed in the Clinical Skills & Simulation Center at our institution as part of a mandatory psychiatry boot camp for fourth-year students matched into a psychiatry residency. The aim was to assess the clinical skill of recognizing a patient requiring emergent care and initiating evaluation and management. The desired learning outcome was to increase graduate students’ experience and confidence in recognizing and managing a patient needing opioid overdose resuscitation outside the hospital setting.

Learners were instructed on out-of-hospital opioid overdose resuscitation during the third-year psychiatry clerkship in a didactic that provided an overview of the opioid crisis and focused on recent clinical guidelines on opioid risk mitigation and prevention and treatment of opioid overdose [14]. The didactic was informed by clinical guidelines from the CDC, the American Heart Association, and the Substance Abuse and Mental Health Services Administration [6,7,17]. The didactic also focused on how medical providers can educate patient and caregivers about recently approved non-prescription formulations of naloxone, now available in most states, and how to reverse an acute opioid overdose.

The learners were graduating fourth-year students matched into a psychiatry residency. All learners in 2017-2018 and 2018-2019 completed a feedback questionnaire after the OSCE, and associated debriefing took place. In 2018-2019 an additional formative assessment of overdose resuscitation was added in the third year, and a brief refresher didactic was added in the Psychiatry Boot Camp.

The questionnaire assessed to what degree learners agreed that the OSCE “was realistic and believable” and “was valuable as an educational tool,” and inquired how confident students felt after completion of this opioid OSCE station and the debriefing session in their clinical skill in managing out-of-hospital treatment of acute opioid overdose. The agreement/confidence level was measured on a 5-point Likert scale (1 = not confident at all, 3 = neutral, and 5 = very confident). Learners were also provided with a free-response question to list any suggestions to improve the quality of this opioid OSCE station. The IRB determined that this activity was not research involving human subjects as defined by DHHS and FDA regulations (IRB number: 00001718). 

Equipment

The following equipment was utilized for implementation of this OSCE: 1) High Fidelity Patient Simulator (mannequin), preferably with the ability to constrict pupils and turn lips blue, 2) Mat, 3) Ventilator bag-mask, 4) Plastic mask, 5) IM autoinjector of naloxone simulator or naloxone nasal spray simulator. The student is told a stethoscope and sphygmomanometer are on board in the plane’s emergency medical kit but, to save time in the scenario, the pulse and blood pressure results are provided to the student.

Personnel

A standardized patient (SP) portrayed the patient’s sister, who provides information regarding the patient’s presentation and history. A faculty member served the minimal role of the flight attendant who can communicate between the student and the pilot as needed; this faculty member also led the post-encounter individual debriefing session. A simulation staff member watched the encounter by live video to control the mannequin’s respirations, pulse, pupil size, eye blinking, and colour of lips.

A faculty member served the minimal role of the flight attendant who can communicate between the student and the pilot as requested by the student; this faculty member also led the post-encounter individual and group debriefing sessions and needs to have knowledge and skill in opioid overdose resuscitation. During the two years of use of this OSCE, the same faculty member participated in all of the sessions; he was the Psychiatry Clerkship Director who had taught the third-year clerkship didactics on opioid risk mitigation and opioid overdose resuscitation, and he also directed the Psychiatry Boot Camp.

Implementation

Please see Appendix -A for the detailed simulation scenario. This OSCE was performed in a room in the Clinical Skills & Simulation Center arranged with rows of chairs with a central aisle simulating passenger seating in an aeroplane. Before entering the scenario room, learners were instructed that “You have just graduated from medical school and are flying home for vacation. Midway in the flight, the pilot asks if there is a health care professional on board, and no one else speaks up. You identify yourself to the flight attendant as a graduated medical student. The flight attendant takes you to the back of the plane where a woman is bending down and desperately trying to waken a man lying on the floor to the side of her. The flight attendant has left a ventilator bag-mask as well as a plastic mask in case you need it. Your task is to assess and manage this patient in the next 15 minutes. You will be assessed on your communication and clinical skills. No Post-Encounter Note is needed. At the end of the encounter, you will be debriefed.”

As detailed in Appendix A, the SP immediately tells the student she believes her brother overdosed on hydrocodone. From here, the SP provides additional history as prompted by the student. The student’s assessment of the patient (mannequin) reveals unresponsiveness, cyanotic lips, pinpoint pupils, and a regular pulse of 60 beats per minute but no respirations. The student should instruct the flight attendant to tell the pilot that there is a medical emergency and if possible, the plane needs to land and access EMS.

At the time the student begins to administer artificial breathing, the SP offers the student a simulated IM autoinjector of naloxone (or simulated naloxone nasal spray bottle); she was instructed in its use by the patient’s primary care doctor, and she has carried it in her purse the last 2-3 months in case her brother had an overdose event. The student either administers the naloxone or tells the SP to do so. The faculty observes to see if the student performs the specific steps of resuscitation as outlined in Appendix A. If the proper steps are taken, the mannequin shows a response to CPR and naloxone by eye blinking, resolution of cyanosis, and resumption of respiration at 12 breaths per minute. However, after 3-5 minutes, the patient again becomes unresponsive and stops breathing. The student should recognize this and administer a second naloxone dose or tell the SP to do so. The student should then continue to assess the patient while waiting for the plane to land and the arrival of EMS.

Assessment 

The facilitator reviewed the completion of the critical learner actions list (Appendix A). This list of expected learner actions was derived from a mnemonic of opioid overdose resuscitation, “ACNS” (Assess & Activate EMS; Cardiopulmonary Resuscitation; Naloxone; Stay), as detailed in an earlier publication [14]. Learners received formative feedback following the case during the debriefing, which lasted 10 minutes.

Debriefing

Immediately following the student’s completion of the OSCE, the faculty member remained in the room and provided the student detailed, individualized feedback on his/her completion of each of the learner actions from the critical actions checklist. This debriefing session lasted 10-15 minutes and also included time for open questions and answers about opioid overdose resuscitation and the OSCE scenario. The student then left the room and confidentially and anonymously completed the one-page “Student Feedback on Opioid Overdose OSCE station”, which was later collected during a short group debriefing with all participating students. This group debrief lasted 5 to 10 minutes and was open for general discussion of the topic of opioid overdose resuscitation and review of the American Heart Association guidelines for healthcare provider adult cardiac arrest algorithm [6,17]. 

## Results

In 2017-2019, graduating students entering a Psychiatry residency (N=10) gave positive feedback on the OSCE station. All agreed it “was realistic and believable”, and all agreed (N=6) or strongly agreed (N=4) it “was valuable as an educational tool.” Across 2017-2019, most students agreed (N=7) or strongly agreed (N=1) that they felt confident of their skill in managing out-of-hospital opioid overdose. In contrast, two students reported they were neutral in their confidence in this clinical skill.

## Discussion

The opioid crisis is a public health emergency [3], and medical students and residents may well encounter opioid overdose patients both inside and outside hospital settings. There is an associated need to strengthen undergraduate and graduate medical training on recognition and treatment of opioid overdose, and on educating family and caregivers of patients at risk for such overdose. New guidelines inform this training and education, [4-7,17] and focus on prompt resuscitation that includes naloxone.

To our knowledge, no OSCE to date has been published regarding medical student performance of opioid overdose resuscitation. In this educational summary report, we present an OSCE assessing clinical skills of opioid overdose resuscitation as recommended by current guidelines that were detailed in a self-learning module published by one of the primary authors [14].

We aimed to develop a competency-based assessment for clinical skills in opioid overdose resuscitation outside of the hospital setting. Graduating medical student feedback from 2017-2019 on this Opioid OSCE activity was positive, with all students (n=10) agreeing or strongly agreeing it was valuable as an educational tool, and most felt confident of their skill in managing out-of-hospital opioid overdose.

Though the use of this OSCE was initiated with graduating medical students entering Psychiatry residencies, it could also easily be used in the assessment of medical students entering many other primary cares and speciality fields. It could be used in graduate and continuing medical education as well.

Finally, at our institution during education and training on pain management and appropriate use of opioids, we emphasize “Do not forget the family!” Although medical knowledge and skill are essential in the management of life-threatening opioid overdose, the ability to communicate effectively with the patient’s family members is also essential. This OSCE introduces the engagement of family members during an acute resuscitation, but full education of the family was beyond its scope. The prevention of fatal opioid overdoses involves educating the friends and family of patients as they often serve as immediate first-responders in their communities [12]. This education is especially important for marginalized populations, including minority populations and justice-involved populations, that are disproportionately affected by opioid use disorder [19]. Naloxone can be acquired by community members without a prescription in many states and is easy to administer when first-responders are properly informed on how to do so [12].

Limitations

A significant limitation to this study of the OSCE’s effectiveness is the small number of students studied (n=10). This may have impacted our results, which, therefore, cannot be generalized to other groups. Another limitation is that the primary outcomes were survey items assessing the self-reported level of agreement or confidence with various statements instead of more objective measures. Furthermore, we only provided the students with the questionnaire after the OSCE. However, future studies could provide the questionnaire both before and after the OSCE to better quantify outcomes of this OSCE. One limitation of this OSCE is its dependence on a high-fidelity simulator. However, this simulation could be adapted for implementation with a simple mannequin; for example, the faculty member in the room could inform the student of changes in the patient’s responsiveness, pupil size, etc. over time. 

## Conclusions

Our findings suggest medical trainees benefit from this OSCE skill station and that perhaps this OSCE should also be available to graduating students pursuing other specialities besides Psychiatry. Although designed initially for medical students, this skill station could also be a valuable assessment for residents in various specialities.

## Appendices

Appendix A

Simulation Case Scenario: Opioid Overdose Outside-the-Hospital

Learning Outcome/Objectives:  Use of this simulation OSCE initiated with graduating fourth-year medical students, but its use could be expanded as detailed by the authors. The desired learning outcome is to increase the learner’s experience and confidence in recognizing and managing a patient needing opioid overdose resuscitation outside the hospital setting as outlined in a mnemonic of opioid overdose resuscitation, “**ACNS**”:

**A**ssess and Activate Emergency Medical Services 

**C**ardiopulmonary Resuscitation

**N**aloxone administration

**S**tay and continue to assess until Emergency Medical Services arrive

By the end of this session, learners will be able to:

1. Perform an assessment of a nonresponsive patient with a suspected opioid-related emergency outside the hospital setting.

2. Identify the opioid emergency and activate Emergency Medical Services.

3. Institute the steps of opioid overdose resuscitation including naloxone administration.

4. Continue to assess the patient-and if necessary administer additional naloxone-until Emergency Medical Services arrive.

Overview:  The learner has just completed his/her fourth year of medical school and is flying home for vacation.  Midway in the flight, the pilot asks if there is a health care professional on board, and no one else speaks up.  The learner identifies himself/herself to the flight attendant as a graduated medical student.  The flight attendant hurriedly takes the learner to the back of the plane where a woman is bending down in the aisle desperately trying to waken a man lying on the floor to the side of her.  The flight attendant has left a ventilator bag-mask, as well as a plastic mask, in case they are needed.  The woman says, “Please help me, I think my brother may have accidentally overdosed!  I told him to stop taking so many hydrocodone!” 

The aim is to assess the learner’s clinical skill in recognizing a patient requiring emergent care and initiating evaluation and proper management of opioid overdose.

**Critical Actions** for the Learner: 

1. Perform an assessment of a nonresponsive patient with a suspected opioid-related emergency outside the hospital setting.

2. Recognize a life-threatening opioid emergency and activate Emergency Medical Services.

3. Institute the steps of opioid overdose resuscitation including naloxone administration.

4. Continue to assess the patient-and if necessary administer additional naloxone-until Emergency Medical Services arrive.

Learner Instructions (these written instructions are placed on the door outside the simulation room):

Patient:  Jim

Age:  30

Setting/Chief Complaint:  You have just graduated from medical school and are flying home for vacation.  It is midway in the flight, and the pilot asks if there is a health care professional on board, and no one else speaks up.  You identify yourself to the flight attendant as a graduated medical student, and you are hurriedly taken to the back of the plane where a woman is bending down in the aisle desperately trying to waken a man lying on the floor to the side of her.  The flight attendant leaves a ventilator bag-mask as well as a plastic mask in case you need it. A stethoscope and sphygmomanometer are onboard in the plane’s emergency medical kit, and you obtain the following vital signs:

Heart Rate/minute:  60 beats per minute, regular rhythm

Blood Pressure:  108/70 mm Hg

Respiratory Rate/minute:  0 breaths/minute

TASK (15 minutes):  Assess and manage this patient.  You will be assessed on your communication and clinical skills. 

No Post-Encounter Note is needed.  At the end of the encounter you will have a post-encounter debriefing session.

_____________________

Physical setting:  This OSCE was performed in a room arranged with rows of chairs with a central aisle simulating passenger seating in an airplane. A standardized patient (SP) is bending down from an aisle chair desperately trying to waken a man (mannequin) lying supine on the aisle floor to the side of her. 

Beginning of Scenario:  The SP immediately tells the learner “Please help me, I think my brother may have accidentally overdosed! I told him to stop taking so many hydrocodone!” From here, the SP provides additional history only as prompted by the learner. A sole faculty member in the room serves the minimal role of the flight attendant who can communicate between the student and the pilot as needed (this faculty member also leads the post-encounter debriefing sessions).

Jim's sister (SP) provides additional history only as prompted by the student: 

History of Present Illness:  Jim is a 30-year-old male taking hydrocodone-both prescribed and from street purchase-for chronic low back pain.  He was sitting in an aisle seat on an airplane mid-flight when his sister noticed him poorly responsive and gasping for air; when Pat shook Jim to try to stir him, Jim fell into the aisle of the airplane passenger cabin.

Jim has never been in psychiatric treatment; there is no history of mental health problems except the SP is concerned that Jim has been increasing his use of street-purchased hydrocodone.  

Past Medical History: 2-year history of low back pain dating to strain in lifting a washing machine. No history of other medical problems, and no hospitalizations or surgeries.

Medications:  Hydrocodone 325 mg/Acetaminophen 325 mg, two oral tablets every 6 hours as needed for pain. Jim's sister (the SP) is certain Jim has been purchasing hydrocodone from the street in recent months to supplement prescription hydrocodone.

Allergies:  none

Social History:  Unremarkable developmental milestones; high school graduate. No military or marital history; the remote history of arrest for cannabis possession. Single male; works for furniture company but job assignment changed to clerical work due to his chronic back pain.  Lives alone in apartment.

Tobacco:  1 pack per day. 

Alcohol:  3 beers on Saturdays and Sundays.

Illicit drugs:  Jim used cannabis in teen years, including daily use from age 18 to 20 years old. 

Family history:  Father: hypertension. Paternal uncle: opioid use disorder.

Physical Exam:  The learner’s assessment of the patient (mannequin) reveals: 

HEENT:   Lips are cyanotic.  Pupils are pinpoint.

Lungs:  No respirations.

Cardiovascular:  Pulse: 60 beats per minute, regular rhythm.

Neurological:  The patient is unresponsive to voice, shake, and sternal rub.

____________________

Interventions/Timeline:

At approximately 1 minute: The patient (mannequin) is not breathing, and the learner should have initiated rescue breathing. If the learner had requested an automated external defibrillator at this time the flight attendant hands the learner an index card with the following information: “The automated external defibrillator has arrived and reveals ‘No Shock Indicated’. The automated external defibrillator remains attached throughout the rest of this scenario.” The flight attendant also informs the learner that the plane’s emergency medical kit does not include naloxone. The SP offers the learner an intramuscular autoinjector of naloxone; as the closest family member of Jim, she was instructed how to use the naloxone injector by Jim’s primary care physician, and she has carried it in her purse the last 2-3 months in case Jim had an overdose event

At approximately 4 minutes:  This is 2 minutes after the learner or the SP administers the naloxone dose intramuscular by autoinjector. The patient (mannequin) begins to breathe spontaneously at a rate of 12 breaths/minute.  If possible the bluish color of the lips resolves and the mannequin should also begin to have some eye blinking but no verbalization. The patient’s pulse remains at 60 bpm with a regular rhythm throughout the entire scenario.

At approximately 9 minutes:  This is 3-5 minutes after the patient’s respirations had returned. The patient (mannequin) again becomes unresponsive and stops breathing. The learner should again initiate rescue breathing at 12 breaths/minute while also administering a second dose of naloxone IM by autoinjector into the patient’s other upper thigh.  If the learner asks, the automated external defibrillator continues to indicate “No Shock Indicated.”

At approximately 12 minutes:  This is 2 minutes after the learner or the SP administers the second naloxone dose intramuscular by autoinjector. The patient begins to have responsiveness-eye blinking but no verbalization-and resumes breathing at a rate of 12 breaths/minute. The learner continues to assess the patient until the session is ended at approximately 15 minutes.

_____________

Ideal Scenario Flow

**ASSESS & ACTIVATE:**  The learner enters the room and finds the unresponsive patient (mannequin) supine on the floor with his sister (the SP) leaning over him and shaking him. The flight attendant (faculty member) is standing nearby.  The learner quickly confirms the patient is unresponsive to shouting/shaking/sternal rub and is not breathing but there is a pulse of 60 beats per minute.  The SP quickly tells the learner that she suspects a hydrocodone overdose. The learner should assess the patient for additional signs of opioid overdose such as the color of the lips or fingernails, size of the pupils, as well as for signs of head trauma. The learner should tell the flight attendant to inform the pilot that this is a 911/Emergency Medical Services situation. The learner also asks the attendant to bring any emergency medical kit on board including any naloxone and an automated external defibrillator if available. 

**CARDIOPULMONARY RESUSCITATION:**  The patient is unresponsive with no breathing; the learner should initiate rescue breathing with 1 breath every 5 seconds (12 breaths/min).  The learner should recheck the pulse about every 2 minutes. The pulse remains 60 bpm throughout the OSCE.  The flight attendant hands the learner an index card with the following information: “The automated external defibrillator has arrived and reveals ‘No Shock Indicated’. The automated external defibrillator remains attached throughout the rest of this scenario.”

**NALOXONE:**  The SP indicates she has an intramuscular autoinjector of naloxone in her purse.  The learner should briefly stop rescue breathing and administer the naloxone in the anterolateral aspect of the patient’s thigh--through clothing if necessary-or have the SP do so.  Subsequently, when the patient blinks and resumes spontaneous breathing at a rate of 12 breaths/minute, the learner stops rescue breathing but explains to the SP she needs to continue to closely monitor the patient.  Several minutes later the patient again stops breathing, and the learner either gives a second intramuscular autoinjector dose of naloxone, or tells the SP to do so, and the learner resumes rescue breathing. The automated external defibrillator continues to indicate “No Shock Indicated.”

**STAY:**  Subsequently the patient again has eye blinking and resumes breathing at 12 breaths/minute. The learner discontinues rescue breathing, but remains with the patient with plans to monitor him until the plane can land and care can be turned over to Emergency Medical Services. The learner explains to the SP what has transpired and answers her questions.

_____________

Anticipated Management Mistakes:

Failure to recognize the lack of indication for chest compressions:  We found that sometimes the learner initiated both rescue breathing and chest compressions, at times due to difficulty detecting the pulse on the mannequin (despite a pulse being generated by the high fidelity simulator mannequin).  We modified our sessions such that if the learner begins chest compressions, the faculty member in the room informs the learner that the patient has a pulse of 60 bpm.
